# Juvenile Atlantic cod behavior appears robust to near-future CO_2_ levels

**DOI:** 10.1186/s12983-015-0104-2

**Published:** 2015-05-23

**Authors:** Fredrik Jutfelt, Maria Hedgärde

**Affiliations:** Department of Biological and Environmental Sciences, University of Gothenburg, PO Box 463, SE-405 30 Göteborg, Sweden; The Lovén Centre Kristineberg, Kristineberg 566, SE-451 78 Fiskebäckskil, Sweden

**Keywords:** Carbon dioxide, Teleost, Climate change, Boldness, Teleost, Lateralization, Behavior, *Gadus morhua*, Ocean acidification

## Abstract

**Background:**

Ocean acidification caused by the anthropogenic release of CO_2_ is considered a major threat to marine ecosystems. One unexpected impact of elevated water CO_2_ levels is that behavioral alterations may occur in tropical reef fish and certain temperate fish species. These effects appear to alter many different types of sensory and cognitive functions; if widespread and persistent, they have the potential to cause ecosystem changes.

**Methods:**

We investigated whether economically and ecologically important Atlantic cod also display behavioral abnormalities by exposing 52 juvenile cod to control conditions (500 μatm, duplicate tanks) or an end-of-the-century ocean acidification scenario (1000 μatm, duplicate tanks) for one month, during which time the fish were examined for a range of behaviors that have been reported to be affected by elevated CO_2_ in other fish. The behaviors were swimming activity, as measured by number of lines crossed per minute, the emergence from shelter, determined by how long it took the fish to exit a shelter after a disturbance, relative lateralization (a measure of behavioral turning side preference), and absolute lateralization (the strength of behavioral symmetry).

**Results:**

We found no effect of CO_2_ treatment on any of the four behaviors tested: activity (F = 1.61, p = 0.33), emergence from shelter (F = 0.13, p = 0.76), relative lateralization (F = 2.82, p = 0.50), and absolute lateralization (F = 0.80, p = 0.26).

**Conclusion:**

Our results indicate that the behavior of Atlantic cod could be resilient to the impacts of near-future levels of water CO_2_.

## Introduction

The accelerating rate of anthropogenic emissions of CO_2_ [1] results in higher oceanic surface pCO_2_ and lower pH in a process known as ocean acidification. For the 650,000 years preceding the Industrial Revolution, the CO_2_ concentration in the atmosphere did not exceed 300 ppm [2]; however, the atmospheric levels are now at 400 ppm and may reach close to 1000 ppm by the end of the current century according to the fossil fuel intensive IPCC RCP8.5 emission scenario [1]. Numerous studies have investigated the effect of ocean acidification-like CO_2_ exposure on invertebrates [3], and the responses differ dramatically between groups, species, environments and life stages [4]. In contrast, fewer studies have investigated the possible effects of ocean acidification on fish because they have long been considered among the most CO_2_-tolerant marine organisms [5-7].

However, a number of reports in recent years have suggested that the behavior of coral reef fish, including their activity level, boldness, behavioral asymmetry (lateralization), and responses to olfactory and auditory cues, may be affected by ocean acidification (see the review by [8]). These behavioral effects appeared at ocean acidification-relevant levels of CO_2_ (700–1200 μatm). Behaviors such as foraging, competition and predator avoidance are important in many aspects of the lives of fish, and any disruption of normal behavior is expected to affect fitness [8]. A switch from repulsion to attraction to the scent of predators has been reported in several coral reef fish species after CO_2_ exposure [8,9]. Juvenile leopard coral grouper (*Plectropomus leopardus*) exposed to 965 μatm CO_2_ was reported to dramatically increase spontaneous activity and decrease shelter usage, a pattern of behaviors that is indicative of hyperactivity and/or increased boldness [10]. In addition, several species of damsel fish and cardinal fish living near natural volcanic CO_2_ seeps were reported to show the same behavioral effects on olfaction, activity and boldness, which may indicate that the effect is not restricted to laboratory CO_2_ exposure [11].

Lateralization is the asymmetry of brain function manifested as asymmetrical behavior, and behavioral lateralization has recently been shown to be significantly reduced by CO_2_ exposure in the coral fish *Neopomacentrus azysron* [12] as well as in the temperate three-spined stickleback (*Gasterosteus aculeatus*) [13,14]. Relative behavioral lateralization is the left- or right-side preference of an individual or a population in a choice situation, and absolute behavioral lateralization is the strength of that side bias. Various fitness advantages have been correlated with lateralized (showing a side preference) behavior [15,16], and stronger lateralization correlates with increased escape reactivity. This observation suggests that lateralization may confer a fitness advantage through an increased ability to escape from predator attacks [15], but causality has yet to be demonstrated.

Recently, it was suggested that the reversal of lateralization and olfaction is related to a reversal of GABA_A_ receptor function [14,17]. Most inhibitory synapses in the brain of vertebrates involve the neurotransmitter GABA [18], and it has been suggested that a decrease in plasma Cl^−^ and an increase in HCO_3_^−^ can disrupt the hyperpolarization function of the GABA_A_ receptor, which may become dysfunctional or excitatory. This reversal of GABA_A_ function could explain the shifts and, in some cases, reversals in behavior [14,17].

Although it has been reported that a number of Australian coral reef fishes demonstrate altered behavior when exposed to CO_2_, there is fragmented knowledge regarding CO_2_-induced behavioral shifts in species from other parts of the world. Adult three-spined sticklebacks were reported to display altered behavior following long-term exposure to 990 μatm CO_2_ [13], and the phototactic response in newly hatched larvae of temperate two-spotted goby (*Gobiusculus flavescens*) was increased by CO_2_ exposure [19]. Furthermore, temperate Pacific splitnose rockfish (*Sebastes diploproa*) increased their time spent in darkness when exposed to elevated CO_2_ levels [20], indicating reduced boldness in contrast to the increased boldness reported in coral reef fish. Other temperate fishes have been suggested to be less affected by elevated pCO_2_ [21,22].

Atlantic cod (*Gadus morhua*) is an ecologically and economically important species that has a history of being exposed to overfishing [23] and cod populations may therefore be sensitive to the effects of additional stressors such as ocean acidification. It has been suggested that large juvenile cod are physiologically tolerant to very high CO_2_ levels, maintaining swimming capacity and aerobic scope despite long exposure. Increased gill Na^+^/K^+^-ATPase activity has been suggested as an acclimation mechanism to improve the capacity for gill ion transport [6]. In contrast to their high physiological acclimation capacity, Frommel et al. [24] demonstrated that Atlantic cod larvae could suffer severe and, most likely, lethal tissue necrosis when exposed to 1800 and 4200 μatm CO_2_. Larvae from the same experiment were also investigated for behavioral changes using a 3-D tracking system, but major deviations from normal behavior were not observed despite the very high pCO_2_ [25]. Post-settlement juveniles, however, exhibit a different behavioral gamut than pelagic larvae, including benthic exploration and interaction with the substrate, as well as shelter use [26-28]. This makes the juvenile stage suitable for testing behaviors in specific arenas, such as lateralization double T-chambers, activity tanks and emergence from shelter tanks.

Therefore, the aim of the present study was to investigate whether juvenile Atlantic cod show alterations in these specific behaviors when exposed to elevated CO_2_ levels. Based on the behaviors affected by exposure to elevated CO_2_ levels in coral reef fish and sticklebacks, three different behavior experiments were performed in control and high CO_2_-exposed cod: activity trials, emergence from shelter and lateralization.

## Materials and methods

### Fish rearing and treatment

The ethical committee for animal experimentation approved all experiments (Gothenburg, Sweden, ethical permits Jutfelt 100–2010 and Jutfelt 151–2011).

The fish (56 juvenile Atlantic cod) were captured by Kristineberg Marine station (lat. 58.249717, long. 11.445522), Lysekil, Sweden, with hand nets, seine nets and fish trap cages. Length and weight was measured for each individual at the start of the experiment. Fish were randomly distributed to four tanks, two for each treatment. All experiments were conducted in thermally constant rooms that ensured temperature stability at all times. The light regime was L16:D8 h.

Four dark cylindrical fiberglass 100 L aquaculture tanks with coned bottoms (Strandvik Plast AS, Strandvik, Norway) were continuously supplied with flow-through deep water that was continuously pumped from 30 meters depth in the fjord. Each fish tank was supplied with flow-through water at 3 L per minute from its own 200 L header tank, which were heavily aerated and in two of the header tanks the CO_2_ levels were manipulated. The fish were thus exposed to control water (532 μatm ± 43 SD) or water with elevated CO_2_ levels (1014 μatm ±76 SD) for the duration of the 30 day exposure period. The exposure duration was chosen to presumably allow acclimation, as acclimation to other environmental stressors such as temperature occurs over days to weeks [29]. The fish tanks were covered with clear plastic lids to reduce gas exchange. The pCO_2_ of the fish tanks was measured at least once daily using an infrared pCO_2_ measurement system, a Vaisala GM70 (Vaisala, Helsinki, Finland) connected to a gas-permeable silicone membrane according to [30-32]. pH-stat computers (Aquamedic, Bissendorf, Germany) controlled the pH of the two CO_2_-manipulated header tanks by bubbling of pure CO_2_ (Aga gas, Gothenburg, Sweden) through solenoid valves. The temporal pH variance of the header tanks was low (<0.1 pH units), and the pH variability of the fish tanks was negligible (<0.05 pH units). Temperature and salinity were recorded continuously, with a mean temperature of 14.4°C ± 0.44 (SD) and a mean salinity of 33.1 ± 0.8 (SD) PSU. The alkalinity of this deep water supply was very stable and titrated weekly. Oxygen saturation in the fish tanks was always above 90%. The carbonate chemistry was calculated in CO2calc (Hansen, USGS, USA) using data for pCO_2_, salinity, temperature, and alkalinity. The results are shown in Table 1.

**Table 1:** **Water chemistry for the treatments Control and Elevated CO**_**2**_

**Parameter**	**Control**	**Elevated CO**_**2**_
pCO_2_ (μatm)	532.4 ± 42.7	1013.5 ± 76.0
Alkalinity (TA)	2350 ± 37.1	2363 ± 53.7
Salinity (PSU)	33.1 ± 0.8	33.1 ± 0.8
Temp (°C)	14.4 ± 0.5	14.4 ± 0.5
pH_tot_ (calc.)	7.95 ± 0.04	7.69 ± 0.03
Ωaragonite (calc.)	2.10 ± 0.21	1.22 ± 0.08
Ωcalcite (calc.)	3.29 ± 0.33	1.90 ± 0.13

Temperature, salinity, pCO_2_ and alkalinity (A_T_) are measured data; pH_tot_, Ω_aragonite_ and Ω_calcite_ were calculated data using CO2calc (USGS, USA). The data are presented as the means ± SD.

The cod were fed shrimp daily, and any mortality was counted. The fish were not individually tagged because they were to be released after the experiment. The starting lengths and weights, as well as the mortality in the tanks, are presented in Table 2. As mortality occurred in all tanks, it was not possible to determine the growth rates. Half of the mortality was due to cannibalism; a few fish escaped, and the rest died of unknown causes without obvious pathology. After the behavioral tests, the fish were kept for chemosensory evaluation in a related experiment, the results of which have been published in Jutfelt and Hedgärde [31]; thereafter, the fish were released back into the fjord at the site of capture.

**Table 2:** **Tank means of the initial lengths and weights of the fish**

	**Control tank A**	**Control tank B**	**CO**_**2**_ **tank C**	**CO**_**2**_ **tank D**
Length (cm)	8.73 ± 0.18	9.20 ± 0.46	9.44 ± 0.31	10.72 ± 0.54
Weight (g)	5.76 ± 0.34	7.52 ± 1.48	8.37 ± 0.95	13.02 ± 2.38
Mortality (%)	17	11	15	33

The mortality is the total tank mortality over the 30-day exposure in percentage of the starting number of fish in the tank.

From exposure day 12 and onwards, the fish were used in behavioral tests. The test fish were randomly sampled from the tanks, and most of the fish were used in all trials. All tests were performed during the daytime, at the treatment water pCO_2_, temperature and light conditions to which the fish were acclimated. The water in the test chambers was taken from the respective treatment tanks and replaced between trials to maintain water quality and pCO_2_. The pH of the trial water did not differ from pH in the treatment tanks. All trial tanks were visually shielded from disturbance.

### Activity trials

The activity trials were performed during day 12 to 19 after exposure initiation. The activity arena was a 50x50 cm Plexiglas tank with a painted grid bottom forming nine equal squares, and the water depth was 15 cm. The tank was visually shielded from disturbance. A video camera positioned above the tank was used to record the experiments. Single fish were placed in the tank and filmed for one hour, of which the first 30 minutes were considered the acclimatization time. During the remaining 30 minutes, the activity was measured as the number of lines crossed, similar to the activity experiment performed by Munday et al. [10]. The data are presented as lines crossed per minute.

### Emergence from shelter

The experiment on emergence from shelter was performed after 26 days of exposure. The emergence-from-shelter test is commonly used to estimate fish boldness [33]. The arena used for the emergence-from-shelter experiment was a 50 × 50 cm plastic tank with a rock and plastic algae shelter (10 × 10 cm) in one corner, and the water depth was 15 cm. A video camera positioned above the tank was used to record the experiments. Each fish was left to acclimatize to the experimental tank for 5 minutes before it was chased by hand into the shelter. The chasing was short and similar for all fish, and the fish quickly entered the shelter. The time to emerge from the shelter was then measured using the video footage, similar to the methods described in Munday et al. [10].

### Lateralization

Lateralization was tested on days 29 and 30. A double T-maze runway was used for the lateralization trials using the methods described previously [12,13]. The fish were given 5 minutes to acclimatize to the T-maze before starting the trial. The fish were gently encouraged down the central channel using a plastic rod (approaching but not touching the fish) and forced to make a turning decision at each T-crossing. The fish swam spontaneously and did not require much encouragement to follow the central channel and make turning decisions. The procedure was then repeated in the reverse direction. In total, 14 decisions per fish were recorded. The relative and absolute lateralization indices were calculated using:

$$ {L}_r = \left[\left( Turns\ to\  the\  right- Turns\ to\  the\  left\right)\ /\ \left( Turns\ to\  the\  right+ Turns\ to\  the\  left\right)\right]\cdotp 100 $$

and

$$ {L}_a = \left|{L}_r\right| $$

according to [34].

### Data analysis

Statistical analyses were performed in SPSS with a significance level of 0.05. Because all data were normally distributed (Kolmogorov-Smirnov p > 0.05), parametric tests were used. A nested ANOVA (tank nested under treatment) was performed for activity, shelter, relative lateralization and absolute lateralization. When tank effects were found, a post hoc test was used to determine which tanks were significantly different. The data are presented as the mean ± SEM, unless otherwise noted.

## Results

**Figure 1:**
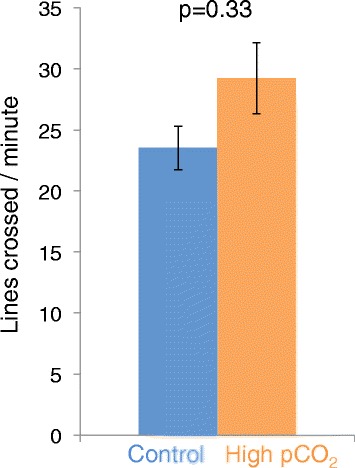
Atlantic cod swimming activity in control and high CO_2_ water. Mean activity levels of Atlantic cod measured as lines crossed per minute during a 30-minute period. The fish were exposed to either control water or high-pCO_2_ water for 12–19 days prior to testing (n_control_ = 22, n_CO2_ = 23). The data represent the mean ± SEM

In the activity trial, a significant tank effect was observed, with fish from one CO_2_-treated tank exhibiting higher activity than the fish from the other three tanks (p = 0.012). The cause of this increased activity is unknown but could be caused by random size differences, as the higher-activity tank contained two of the largest individuals. However, despite the higher activity in one CO_2_ tank, there was no significant effect of the treatment (Figure 1) (nested ANOVA; F = 1.61, p = 0.332, n_control_ = 22, n_CO2_ = 23).

**Figure 2:**
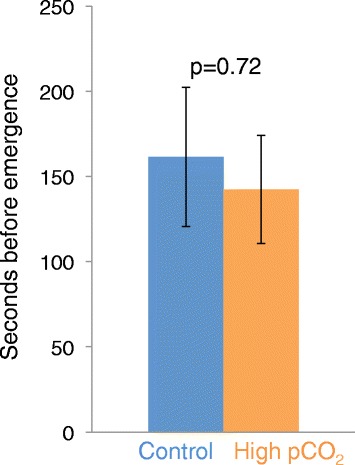
Time to emerge from a shelter in Atlantic cod exposed to control or high CO_2_ water. The mean time in seconds for Atlantic cod to emerge from shelter after being chased, with fish exposed for 26 days to either control water (blue) or high pCO_2_ (orange); n_control_ = 22 and n_CO2_ = 20). The data represent the mean ± SEM.

There was no significant difference in the time to emerge from shelter between the two groups (nested ANOVA; F = 0.13, p = 0.755, n_control_ = 22, and n_CO2_ = 20 (Figure 2), and no tank effect.

**Figure 3:**
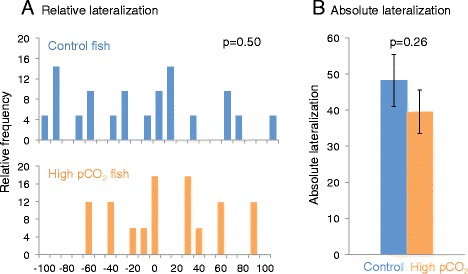
Relative and absolute lateralization in Atlantic cod exposed to control or high CO_2_ water. **A**. Relative lateralization (frequency in %) of Atlantic cod after 30 days of exposure to either control pCO_2_ conditions (blue) or high-pCO_2_ conditions (orange) (n_control_ = 21 and n_CO2_ = 17), where −100 represents 100% left turns and 100 represents 100% right turns. The vertical axis shows the frequency (%). **B**. Mean absolute lateralization; n_control_ = 21 and n_CO2_ = 17). The data represent the mean ± SEM.

There was a tendency for the CO_2_ fish to be right-biased and the control fish to be left-biased, but there was no significant difference in relative lateralization (L_r_) (nested ANOVA; F = 2.82, p = 0.502, n_control_ = 21, n_CO2_ = 17) (Figure 3A). However, there was a significant effect between tanks (p = 0.012). There was no significant difference in the absolute lateralization (L_a_) (nested ANOVA; F = 0.80, p = 0.255, n_control_ = 21, n_CO2_ = 17) (Figure 3B).

## Discussion

Juvenile Atlantic cod and three-spined stickleback have overlapping habitats and temperature ranges, and both species are relatively euryhaline [35]. The recent finding that sticklebacks, similar to a large number of tropical coral reef species, can demonstrate alterations in behavior following the long-term exposure to elevated pCO_2_ could indicate that most teleosts would show these effects. The behaviors tested in this study were chosen as they were previously reported to be affected by CO_2_ exposure in tropical reef fish. In the previous studies, the activity was increased [10], the time until emergence from shelter was reduced [10], and lateralization was reduced [12,17]. In sticklebacks, both lateralization and boldness were found to be affected [13].

However, in the present study on Atlantic cod, none of the behaviors measured were significantly affected by CO_2_ treatment. Although our results differed from those found for juveniles of other investigated species, they were consistent with the data from larval Atlantic cod, in which the larvae were found to be behaviorally tolerant to very high CO_2_ levels [25]. As shown by a related experiment, the dramatic reversal of olfactory preference shown for many Australian coral reef fish was not present in juvenile Atlantic cod [31]. Instead, the juvenile Atlantic cod avoided predator odor to the same high degree (65% of the time in control water), regardless of their exposure water pCO_2_. The lack of behavioral shift in Atlantic cod was somewhat surprising, as many other species have been reported to show abnormal behavior. Atlantic cod are known to forage in hypercapnic deep water [36] and may therefore be physiologically adapted to be tolerant to high environmental CO_2_ levels. These results together suggest that behavioral effects of CO_2_ are not universal in teleosts and that the full geographical and phylogenetic extent of behavioral effects of CO_2_ exposure needs to be elucidated. There are also indications that even within species that appear sensitive to CO_2_-induced behavioral abnormalities, some behaviors are more robust than others. Lateralization in three-spined sticklebacks appear sensitive [13,37], whereas avoidance of bird strikes may be less sensitive or completely resistant [37]. Such differences in sensitivity within species make extrapolations from highly artificial laboratory experiments to impacts on wild populations difficult.

Hyperactivity has been suggested to be an effect of high CO_2_ exposure in fish. The most extreme example reported was in leopard coral grouper, with a 9000% increase in swimming activity, as measured by number of lines crossed [10]. In the present experiment, the high CO_2_ group did not behave significantly differently from the control group. As retrospective power analysis is not recommended [38], we cannot be sure how large effect sizes we could potentially have missed using the current experimental design. However, it is clear that effect sizes of the magnitude reported in many studies on coral reef fish would have been detected by the current experiments.

Some previous experiments have interpreted detected behavioural disturbances as altered function of the central nervous system (CNS) [39], with altered function of inhibitory GABA_A_ receptors implicated as a likely mechanism [17]. However, the finding that fish can acutely detect the water CO_2_ levels and respond to it, even after long term exposure, should not be ignored when designing experiments [31]. It is possible that sensory detection of elevated CO_2_ levels during behavioural trials could alter the behaviour of the fish, for example triggering a search response for better water quality [32]. It can therefore be advisable to include in experimental designs acute CO_2_ exposures, to investigate if such exposures triggers behavioural alterations.

Although we did not find major behavioral disturbances in Atlantic cod, in accordance with other studies [25,31], we cannot rule out effects of elevated CO_2_ of smaller effect sizes, or effects on specific behaviors. However, the behaviors that are highly affected in many coral reef fish appear insensitive to CO_2_ in Atlantic cod (current results and Jutfelt and Hedgärde [31]). Even if the behaviors are robust to elevated pCO_2_, there are nonetheless results showing that future ocean acidification may affect Atlantic cod, including the organ damage shown in Atlantic cod larvae [24]. There is also the potential of ecosystem effects from ocean acidification that could impact Atlantic cod through indirect mechanisms such as interspecific competition [40] or trophic interactions and food quality [41,42].

## Conclusions

The majority of experiments to date on ocean acidification and fish behavior show dramatic effects of elevated CO_2_ levels. This suggests either that most of the fish tested demonstrate abnormal behaviors following high pCO_2_ exposure or that negative results are published to a lower degree, possibly because of researcher or publication biases [43]. Regardless, the results obtained in this study complicate the prediction of future effects of ocean acidification on fish, suggesting that behavioral effects could be negligible in some species and that we might not be able to make good predictions until more species from representative geographical and phylogenetic groups are tested and published.
